# Method for simultaneous voxel-based morphometry of the brain and cervical spinal cord area measurements using 3D-MDEFT

**DOI:** 10.1002/jmri.22340

**Published:** 2010-11

**Authors:** Patrick AB Freund, Catherine Dalton, Claudia AM Wheeler-Kingshott, Janice Glensman, David Bradbury, Alan J Thompson, Nikolaus Weiskopf

**Affiliations:** 1 Department of Brain Repair and Rehabilitation, UCL Institute of NeurologyLondon, United Kingdom; 2 Wellcome Trust Centre for Neuroimaging, UCL Institute of NeurologyLondon, United Kingdom; 3 Department of Neuroinflammation, UCL Institute of NeurologyLondon, United Kingdom

**Keywords:** spinal cord injury, atrophy, cervical cord, cross sectional cord area, MDEFT, validation

## Abstract

**Purpose:**

To investigate whether a 3D-modified driven equilibrium Fourier transform (MDEFT)-based acquisition protocol established for brain morphometry also yields reliable information about the cross-sectional spinal cord area (SCA).

**Materials and Methods:**

Images of brain and cervical cord of 10 controls and eight subjects with spinal cord injury (SCI) were acquired with the 3D-MDEFT-based imaging protocol and an 8-channel receive head coil. The new protocol was validated by two observers 1) comparing the SCA measured with the standard acquisition protocol (3D magnetization-prepared rapid acquisition gradient echo [MPRAGE] and dedicated spine coil) and the new protocol; and 2) determining the scan–rescan reproducibility of the new protocol.

**Results:**

Scan–rescan reproducibility of SCA measurements with the MDEFT approach showed a similar precision for both observers with standard deviation (SD) <4.5 mm^2^ and coefficient of variation (CV) ≤5.1%. Analysis of variance (ANOVA) revealed a main effect of observer and interaction between observer and scan protocol that could be primarily attributed to a small observer bias for MPRAGE (difference in SCA <2.1 mm^2^). No bias was observed for 3D-MDEFT vs. 3D-MPRAGE.

**Conclusion:**

The 3D-MDEFT method allows for robust unbiased assessment of SCA in addition to brain morphology. J. Magn. Reson. Imaging 2010;32:1242–1247. © 2010 Wiley-Liss, Inc.

THE ENDPOINT OF NEURODEGENERATION is atrophy that occurs in the brain and spinal cord. Voxel-based morphometry (VBM) (1) and spinal cord cross-sectional area (SCA) (2) are sensitive noninvasive magnetic resonance imaging (MRI)-based measures with the potential to detect morphometric changes. VBM has been extensively used to investigate volumetric cortical changes associated with disease (3) and in aging studies (4). The 3D modified driven equilibrium Fourier transform (3D-MDEFT) sequence (5,6) was specifically optimized (7) and shown to be optimal for VBM analysis based on T1-weighted (T1w) images (8). However, it is not known if the 3D-MDEFT sequence also provides reliable measures of the SCA. SCA per se has proven to be an important marker of disease progression in multiple sclerosis (MS), reflecting axon/myelin loss (2,9). Few studies have investigated atrophic changes after traumatic spinal cord injury (SCI) in the brain (10) and spinal cord (11). The relative scarcity of investigations aiming at quantifying atrophic changes at the spinal level may be explained by the small size of the cord and the potential artifacts from fractured disk, fixative MRI implants, motion, and cerebrospinal fluid (CSF) pulsation.

Different dedicated techniques were developed that reliably measure the SCA on T1w 3D magnetization-prepared rapid acquisition gradient echo (3D-MPRAGE) images (2,12–15). A semiautomated intensity-based contouring technique, initially developed for MS applications by Losseff et al (2), has been shown to be sensitive in assessing longitudinal pathological changes associated with tissue loss of the cervical spinal cord (2,9). The Losseff method is based on T1w 3D-MPRAGE images acquired with dedicated spine array coils (2). The method segments the spinal cord from the 3D anatomical scan and gives the mean SCA over five 3-mm-thick reformatted axial slices at the C2 spinal level.

More recent studies propose techniques for measuring SCA on 3D-MPRAGE images that address variance and bias in SCA measurements due to partial volume effects, image orientation, and intra- and interoperator variability (2,12–15). One method uses a B-spline active surface model for SCA quantification that also provides additional measures such as SC volume (14), but showed decreased sensitivity in longitudinal studies compared to the Losseff method (12). Recently, another active surface model was developed with additional smoothness constraints that allows efficient measurement of the SCA but shows a relatively high bias of ≈14% compared to the Losseff method (15). The method by Tench et al (12) estimates the partial volume effect from the image intensities at the automatically detected edge between the cord and CSF. In addition to the correction for the partial volume effect, the method also corrects for imperfect orientation of the spinal cord in the images, yielding slightly improved sensitivity of the method compared to the Losseff method (coefficient of variation [CV] = 0.55% vs. 0.79%) and improved accuracy (bias of 3.15% vs. 4.3%) for healthy volunteers (2,12).

Ideally, scan time should be kept as short as possible, especially for patients. At present, the volumetric assessment at spinal and cortical level is derived from two separate acquisitions, one optimized for the spinal cord (2) and the other one for the brain (3D-MDEFT (7)).

We set out to establish an imaging protocol for a fast, comprehensive assessment of volumetric changes at the level of the cervical spinal cord and cortex based on a modified version of a T1w 3D-MDEFT acquisition protocol established for brain morphometry. We simultaneously acquired brain and cervical cord images using a 3D-MDEFT sequence in combination with an 8-channel head coil that provided good coverage of the cervical cord. In a cohort of healthy controls and SCI subjects, we assessed the accuracy and precision of this new technique by comparing it to the established standard 3D-MPRAGE sequence with a dedicated spine coil at the clinical field strength of 1.5T (ie, the Losseff technique (2)).

## MATERIALS AND METHODS

### Subjects

Eight male subjects with traumatic SCI (mean disease duration of 13 years) at level C5/C8 and 10 healthy gender, body height, and weight-matched control subjects were recruited. The mean age of the SCI subjects was 45.1 years (standard deviation [SD] 10.9, range 29–61) and for controls it was 34 years (SD 13, range 25–64). All participants were right-handed.

All participants gave informed, written consent before participating in the study, which was approved by the Joint Ethics Committee of the Institute of Neurology and the National Hospital for Neurology and Neurosurgery.

### Imaging Protocol

All data were obtained on a 1.5T Magnetom Sonata MRI scanner operated with a radiofrequency (RF) body transmit coil (Siemens Healthcare, Erlangen, Germany). The new imaging approach used an 8-channel head receive coil (manufactured by MRI Devices, Orlando, FL; distributed by Siemens) for signal reception and a T1w 3D-MDEFT sequence that is well established for high-resolution brain imaging and brain morphometry (7) with the following parameters: 176 sagittal partitions, 256 × 256 image matrix, field of view (FOV) = 256 × 256 mm^2^, isotropic 1 mm^3^ resolution, TR/TE/TI = 12.24/3.56/530 msec, bandwidth (BW) = 106 Hz/Px, α = 23°, 13 minutes 43 seconds acquisition time. The original implementation of the 3D-MDEFT sequence applied spin tagging in the neck to suppress flow artifacts. Since we used the RF body coil for transmission and nonselective RF pulses, a large area outside the imaging region was saturated including inflowing blood. Therefore, spin tagging was not necessary to reduce inflow artifacts and it was disabled to preserve signal in the cervical spinal cord.

For comparison of the new imaging protocol with the established standard (Losseff technique), a T1w 3D-MPRAGE (16) protocol was scanned with the following parameters according to Losseff et al (2) and using the vendor's standard spine coil array: 60 sagittal partitions, 256 × 256 image matrix, FOV = 250 × 250 mm^2^, 0.98 × 0.98 × 1 mm^3^ resolution, TR/TE/TI = 1300/5.19/450 msec, BW = 130 Hz/Px, α = 20°, acquisition time 5 minutes 30 seconds. To assess the scan–rescan stability of the new technique, two 3D-MDEFT scans were acquired on different days (>1 month apart, range 1–9 months). A 3D-MPRAGE scan was acquired on the same day as the second 3D-MDEFT scan.

For each MDEFT scan the subject's nasion and for each MPRAGE scan the subjects' lips were positioned in the isocenter of the gradient and RF body coil by using the standard laser positioning system. To determine the exact relative position of the C2 disk in the gradient and RF transmit field, the position of the C2 disk with regard to the position of the isocenter of the gradient coil/RF body coil was measured in the z direction (ie, direction along the static B0 field) on the acquired MDEFT/MPRAGE images, respectively.

The subjects' head was fully put into the head coil with the vertex touching the rear end of the coil, maximizing the signal received from the cervical cord. The rostral edge of the FOV was aligned with the vertex and thus with the rear end of the head coil. The exact relative position of the C2 disk in the 8-channel RF receive head coil was determined by measuring the distance from the caudal edge of the C2 disc to the end of the FOV on the acquired MDEFT images. For the SCI subjects and controls the influence of the relative position in the gradient or 8-channel RF receive coil with regard to the SCA (see Image Analysis, below) was assessed by Pearson's correlation with a significance threshold of *P* < 0.05.

For an independent assessment of the accuracy of the new SCA measurement approach, phantoms with acrylic rods with three different cross-sectional areas were filled with doped water (≈3% Magnevist, Bayer HealthCare Pharmaceuticals, Leverkusen, Germany) and scanned with the same MDEFT sequence as the participants. The contrast of the phantom images was inverted to yield a similar appearance as the MDEFT images of the spinal cord (contrast-to-noise ratio [CNR] was estimated to be 2.3 and 3.6, respectively; see also Losseff et al (2)). To assess the potential impact of gradient nonlinearities on the accuracy, MDEFT scans were performed with the superior–inferior (SI) center of the sagittal images coinciding with the isocenter and again shifted 7.5 cm from the isocenter in the SI direction (a realistic distance between the nasion and C2). Each scan was acquired twice for estimating scan–rescan reproducibility.

### Image Analysis

The images were transferred to a Sun workstation (Sun Microsystems, Mountain View, CA) and displayed using the Dispimage display software package (Plummer, Department of Physics, University College Hospitals NHS Trust, London, UK). Two trained observers independently measured the SCA on a series of five contiguous axial slices (3-mm slice thickness) using a semiautomated segmentation as described (2). Briefly, the C2 disc served as the caudal landmark and slices were reformatted perpendicular to the spinal cord. A region of interest (ROI) was drawn around the cord CSF space and the cord itself on each slice. Mean signal intensities in the two ROIs informed a threshold-based automatic segmentation of the cord. The SCA was automatically estimated in each slice and averaged. Intraobserver reproducibility was assessed for the postacquisition analysis step (ie, the SCA was measured twice on the same data with a gap >7 days) and for scan–rescan (ie, from the two separate MDEFT images acquired on different days at least 1 months apart). The postacquisition intraobserver reproducibility analysis was conducted on a randomly chosen subset of five controls only, the scan–rescan reproducibility was assessed on all 18 subjects. The intraobserver SD and CV (averaged by root-mean-square method over the group) were used as measures of the postacquisition analysis and scan–rescan reproducibility (17). Further, the interobserver reproducibility between observer 1 and observer 2 was determined for the MPRAGE and the second MDEFT acquisitions on all 18 subjects.

The dependence of measured SCA on the imaging method (brain and spinal cord MDEFT vs. spinal cord only MPRAGE/Losseff technique) and observer was assessed by a 2 × 2 repeated measures analysis of variance (ANOVA) (SPSS Statistics 17.0, Chicago, IL) with *P* < 0.05 significance threshold. Data were further explored for systematic differences between the new and established imaging approaches using Bland–Altman plots that allow for robust visualization of comparisons of different measurement techniques (18).

The images of the water phantoms with different-sized rods were analyzed in the same way as the images of the human spinal cord. The accuracy and scan–rescan reproducibility of the rod cross-sectional area measurements were determined for the MDEFT images recorded in the gradient isocenter and 7.5 cm away from it.

## RESULTS

**Figure 1:**
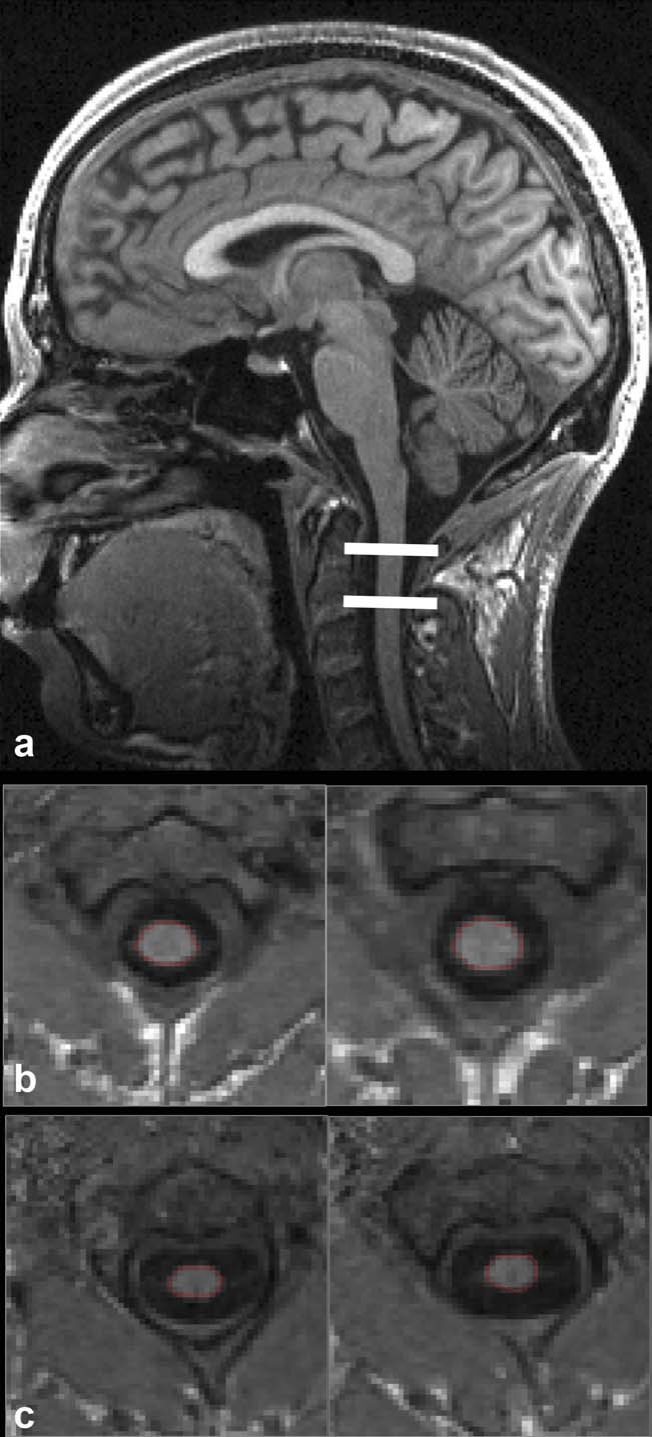
**a:** T1w 3D-MDEFT midsagittal image of a control subject. White lines show location of SCA assessment. **b,c:** Reformatted axial slices at cervical level C2 of the T1w 3D-MDEFT sequences of one control and one SCI subject, respectively. Note the marked reduction of SCA in the SCI subject. The contours (in red) of the SCA defined with the semiautomated technique are superimposed.

The spinal cord was well delineated from the surrounding CSF on the MDEFT images (Fig. 1a) down to the level of C5 due to the MDEFT's intrinsic suppression of signal components with long T1 times, ie, the CSF signal (19). Reduction of SCA in subjects with SCI at cervical level C2 could be detected clearly (see Fig. 1c) compared to controls (Fig. 1b). The mean SCA for control subjects with the MDEFT approach (observer 1) was 81.32 mm^2^ (SD = 5.79 mm^2^) and 81.88 mm^2^ (SD = 5.39 mm^2^) with the MPRAGE protocol. SCI subjects had reduced SCA of ≈38% when compared to controls (MDEFT: 49.25 mm^2^ [SD = 6.7 mm^2^]; MPRAGE: 49.84 mm^2^ [SD = 6.64 mm^2^]).

The intraobserver reproducibility on the same image for the MDEFT/MPRAGE sequence was SD = 1.63 mm^2^ / 0.91 mm^2^, and CV 1.6%/1.5% for observer 1, and SD = 1.62 mm^2^ / 1.3 mm^2^ and CV = 1.7%/1.0% for observer 2. The intraobserver scan–rescan reproducibility of the MDEFT protocol was similar for both observers, ie, SD = 4.49 mm^2^ / 3.48 mm^2^ and CV = 5.1%/3.7% for observer 1 / observer 2. The interobserver SD/CV for the SCA measurement derived from the MDEFT/MPRAGE sequences were SD = 1.65 mm^2^ / 2.12 mm^2^ and CV = 2.3%/3.6%, respectively.

**Figure 2:**
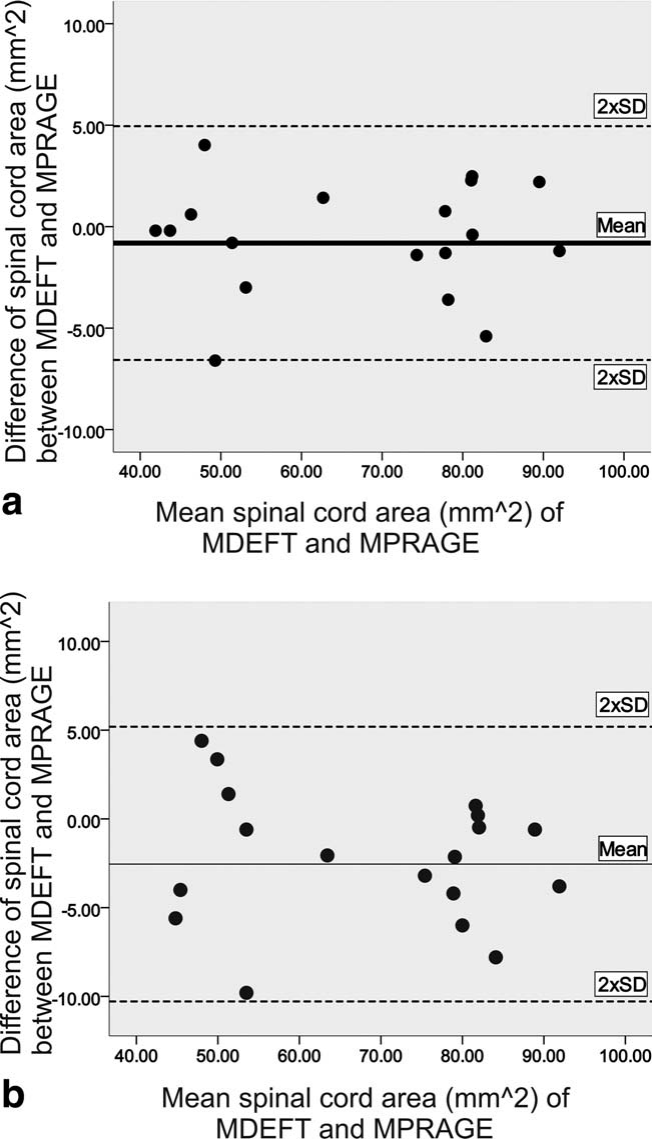
Bland–Altman plots of observers 1 **(a)** and 2 **(b)** assessing the correspondence between the MDEFT and the MPRAGE method for all subjects. No bias or trend in the differences can be observed, indicating good agreement of the two measurement techniques.

The ANOVA revealed a main effect of observer (*df* = 17, F = 5.75, *P* = 0.028) and interaction between observer and scan method (*df* = 17, F = 6.08, *P* = 0.025). Post-hoc paired *t*-tests indicated that the difference could be primarily attributed to a slight observer bias for MPRAGE (*df* = 17, *t* = −2.72, *P* = 0.014). However, the detected bias between observers was minimal (2.01 mm^2^). No bias was detected for the comparison of MDEFT vs. MPRAGE (*P* = 0.4) across observers. Good agreement between the MDEFT and the MPRAGE method for both observers is corroborated by the Bland–Altman plots (Fig. 2), ie, the mean difference between the protocols is well within the 2 × SD confidence intervals. Further, the differences appear to be randomly distributed about the mean and independent of SCA.

No significant correlation between the position of C2 in the gradient or 8-channel receive coil was found (for controls: *P* > 0.18, *r* < 0.31, *n* = 20 [2*10 measurements]; for SCI subjects: *P* > 0.32, *r* < 0.04, *n* = 18).

In the phantom images acquired with the MDEFT sequence, the cross-sectional area of the acrylic rods (and scan–rescan percent deviations) were estimated to be 52.5 mm^2^ (0.4%), 81.3 mm^2^ (0.7%), 131.1 mm^2^ (0.8%) as measured in the gradient isocenter. The areas were 51.9 mm^2^ (1.2%), 78.8 mm^2^ (1.0%), 129.6 mm^2^ (2.2%) as measured 7.5 cm away from the isocenter. The percent deviation from the areas calculated using the diameters measured with a caliber (49.8 mm^2^, 76.9 mm^2^, 132.8 mm^2^, measurement error of ≈3.5%, 2%, 4.2%, respectively) were 5.5%, −1.3%, 5.7% (isocenter), and 4.3%, −2.4%, 2.5% (7.5 cm away from isocenter). From these results it can be seen that the scan position dependent bias in the measured cross-sectional area ranged from 1.1%–3.1% between the two different scan positions.

## DISCUSSION

We have implemented a method for fast and reliable imaging of brain morphology and SCA based on 3D-MDEFT scans with an 8-channel receive head coil (7). The cross-validation with the current established standard based on 3D-MPRAGE scans with a dedicated spine array coil (ie, Losseff et al (2)) showed good agreement in healthy controls and subjects with SCI.

The mean SCA of controls in the present study obtained from the 3D-MDEFT sequences at cervical level C2 (81.32 mm^2^ [SD = 5.79 mm^2^]) was comparable to the values obtained with the standard 3D-MPRAGE (81.88 mm^2^ [SD = 5.39 mm^2^]) in the present study and also in line with previous studies: 82.96 mm^2^ (SD = 8.94 mm^2^) in (20); 81.66 mm^2^ (SD = 2 mm^2^) in (13), the latter study also using a head coil for data acquisition.

The intraobserver and interobserver CV for SCA measurement on the same MDEFT image was ≤2.3%, which is in general agreement with previous reports (2,12,14). The intraobserver scan–rescan CV was ≤5.1% for the MDEFT acquisitions. Although we did not determine the scan–rescan CV for the MPRAGE method in this study, the comparison with previous reports (2,14) suggests an increased CV of the MDEFT method compared to the standard MPRAGE approach (<2.5%). The different CV may be explained by the fact that in our study scan/rescans were performed in two different scan sessions minimally 1 month apart, whereas in some of the previous studies images were acquired within the same scan session (2). Further, the C2 region was relatively far away from the gradient isocenter in case of the MDEFT scans, exacerbating potential gradient nonlinearity effects, although we did not find a significant correlation between the exact C2 position in the RF/gradient coil and SCA. However, phantom measurements indicated a reduction of the measured SCA of ≈3.1% when moving away from the gradient isocenter by 7.5 cm in the SI direction, a realistic distance of C2 from the isocenter. This bias was not observed between the MDEFT and MPRAGE scans performed in vivo, although they were acquired ≈7.5 cm away from each other. Potentially, the bias was masked by a limited precision of the in vivo SCA measurements compared to phantom measurements or compensated by a slightly different contrast of the two acquisition types.

Furthermore, we did not observe an increase of the scan–rescan SD with increasing SCA. Consequently, the CV will be overestimated for populations with a small SCA. Since the volunteers with SCI had a significantly reduced SCA, it is therefore likely that the scan–rescan CV for the MDEFT sequence was overestimated compared to studies with healthy volunteers or MS patients, where significantly larger SCA are typical.

Due to the higher scan–rescan CV, the proposed technique may require larger sample sizes to detect subtle changes over time as observed in other neurodegenerative diseases such as MS (9). However, if larger SCA changes are expected, as for example, observed in the cohort with traumatic SCI, the increased scan–rescan CV is less relevant.

The in vivo comparison between the standard MPRAGE- and new MDEFT-based method indicates a good agreement and no bias between the two approaches. The high accuracy of the MDEFT-based approach is further supported by measurements on phantoms that simulate the spinal cord geometry and showed a bias of less than 5.7% for all phantom diameters and scan positions.

We chose to measure SCA at the C2 level, because the caudal C2 disc offers a well-distinguishable landmark and the spinal cord is well surrounded by the CSF, offering a maximized spinal cord/CSF contrast. Moreover, the intersubject anatomical variability at this level of SCA is known to be small in controls (≈8 mm^2^) (20).

We note that our study design was not aimed at quantifying how much the sequence type/contrast (MPRAGE/MDEFT) or coil type (8-channel/spine) influenced the quality of the SCA measurement. However, the study was designed to and clearly does show that the new combination of MDEFT and 8-channel head coil acquisition achieves unbiased results with similar precision to the standard Losseff et al technique (2). Further studies may investigate what precise impact the different coils or contrasts may have.

The new protocol not only reduces the overall scan time but also provides optimal data for brain morphometry, since the employed 3D-MDEFT sequence has been specially developed to provide optimal gray and white matter contrast in order to perform morphometric measures on brain tissue. Moreover, the particular MDEFT implementation has been optimized for reduced sensitivity to motion, susceptibility artifacts, and B1 field inhomogeneities (7,21). Using VBM, various studies have demonstrated that the MDEFT images allow for detecting subtle GM volume changes (3,4).

In conclusion, the proposed method facilitates the comprehensive assessment of morphological changes in brain and cervical spinal cord. Overall scan time is shortened (14 vs. 19 minutes) and repositioning of the subject and coil adjustment is avoided.

## References

[1] Ashburner J, Friston KJ (2000). Voxel-based morphometry—the methods. Neuroimage.

[2] Losseff NA, Webb SL, O'Riordan JI (1996). Spinal cord atrophy and disability in multiple sclerosis. A new reproducible and sensitive MRI method with potential to monitor disease progression. Brain.

[3] Kloppel S, Stonnington CM, Chu C (2008). Automatic classification of MR scans in Alzheimer's disease. Brain.

[4] Hutton C, Draganski B, Ashburner J, Weiskopf N (2009). A comparison between voxel-based cortical thickness and voxel-based morphometry in normal aging. Neuroimage.

[5] Lee JH, Garwood M, Menon R (1995). High contrast and fast three-dimensional magnetic resonance imaging at high fields. Magn Reson Med.

[6] Ugurbil K, Garwood M, Ellermann J (1993). Imaging at high magnetic fields: initial experiences at 4 T. Magn Reson Q.

[7] Deichmann R, Schwarzbauer C, Turner R (2004). Optimisation of the 3D MDEFT sequence for anatomical brain imaging: technical implications at 1.5 and 3 T. Neuroimage.

[8] Tardif CL, Collins DL, Pike GB (2009). Sensitivity of voxel-based morphometry analysis to choice of imaging protocol at 3 T. Neuroimage.

[9] Stevenson VL, Leary SM, Losseff NA (1998). Spinal cord atrophy and disability in MS: a longitudinal study. Neurology.

[10] Wrigley PJ, Gustin SM, Macey PM (2009). Anatomical changes in human motor cortex and motor pathways following complete thoracic spinal cord injury. Cereb Cortex.

[11] Tuszynski MH, Gabriel K, Gerhardt K, Szollar S (1999). Human spinal cord retains substantial structural mass in chronic stages after injury. J Neurotrauma.

[12] Tench CR, Morgan PS, Constantinescu CS (2005). Measurement of cervical spinal cord cross-sectional area by MRI using edge detection and partial volume correction. J Magn Reson Imaging.

[13] Mann RS, Constantinescu CS, Tench CR (2007). Upper cervical spinal cord cross-sectional area in relapsing remitting multiple sclerosis: application of a new technique for measuring cross-sectional area on magnetic resonance images. J Magn Reson Imaging.

[14] Hickman SJ, Coulon O, Parker GJ (2003). Application of a B-spline active surface technique to the measurement of cervical cord volume in multiple sclerosis from three-dimensional MR images. J Magn Reson Imaging.

[15] Horsfield MA, Sala S, Neema M (2010). Rapid semi-automatic segmentation of the spinal cord from magnetic resonance images: application in multiple sclerosis. Neuroimage.

[16] Mugler JP, Brookeman JR (1990). Three-dimensional magnetization-prepared rapid gradient-echo imaging (3D MP RAGE). Magn Reson Med.

[17] Bland JM, Altman DG (1996). Measurement error proportional to the mean. Br Med J.

[18] Altman DG, Bland JM (1986). Comparison of methods of measuring blood pressure. J Epidemiol Community Health.

[19] Hochmann J, Kellerhals H (1980). Proton NMR on deoxyhemoglobuse of a modified DEFT technique. J Magn Reson.

[20] Rashid W, Davies GR, Chard DT (2006). Upper cervical cord area in early relapsing-remitting multiple sclerosis: cross-sectional study of factors influencing cord size 2. J Magn Reson Imaging.

[21] Howarth C, Hutton C, Deichmann R (2006). Improvement of the image quality of T1-weighted anatomical brain scans. Neuroimage.

